# Effectiveness of Integrative Laughter Therapy to Reduce Anxiety, Improve Self-Esteem and Increase Happiness: A Naturalistic Study at a Day Hospital for Addictive Disorders

**DOI:** 10.3390/ijerph16214194

**Published:** 2019-10-30

**Authors:** Seyla De Francisco, Cristina Torres, Sandra De Andrés, Ana Millet, M. Teresa Ricart, Elvira Hernández-Martínez-Esparza, Mercedes Abades, Joan Trujols

**Affiliations:** 1 Escola Universitària d’Infermeria de l’Hospital de la Santa Creu i Sant Pau (EUI Sant Pau), Universitat Autònoma de Barcelona (UAB), 08025 Barcelona, Spain; mricart@santpau.cat (M.T.R.); ehernandezma@santpau.cat (E.H.-M.-E.); mabades@santpau.cat (M.A.); 2 Unitat de Conductes Addictives, Servei de Psiquiatria, Hospital de la Santa Creu i Sant Pau, 08025 Barcelona, Spain; ctorresa@santpau.cat (C.T.); sandres@santpau.cat (S.D.A.); annamillet1052@gmail.com (A.M.); 3 Institut d’Investigació Biomèdica Sant Pau (IIB Sant Pau), 08025 Barcelona, Spain; 4 Centro de Investigación Biomédica en Red de Salud Mental (CIBERSAM), 28029 Madrid, Spain

**Keywords:** laughter therapy, addictive disorders, day hospital treatment, mental health nursing, self-esteem, happiness, satisfaction with life, anxiety, quasi-experimental design

## Abstract

Little is known about the effectiveness of laughter therapy as an adjunctive treatment for patients with addictive disorders. This study aims to evaluate the benefits of integrative laughter therapy (ILT) on levels of self-esteem, anxiety, and happiness in patients treated for addiction at a day hospital (DH). A prospective, naturalistic study with a pre-post design was conducted. All 185 participants received the standard, multicomponent treatment at the DH (treatment as usual; TAU). The participants were also invited to attend weekly ILT sessions. Upon completion of the 2-month DH treatment program, patients were classified according to their attendance at the ILT sessions: patients who attended ≥80% constituted the experimental group (TAU + ILT group) while those who attended <80% were considered controls. Although both groups achieved statistically significant increases in self-esteem and happiness with a decrease in trait anxiety, the improvement on these variables was significantly greater in the TAU + ILT group. Subject to the limitations inherent to quasi-experimental research, the findings of the present study suggest that the addition of an ILT module to the standard treatment in a DH for addictive disorders may yield greater improvement in self-esteem, anxiety, and happiness compared to TAU.

## 1. Introduction

Several recently published systematic reviews have demonstrated the beneficial—or, at least, promising—impact of laughter and humour interventions for a wide range of conditions and populations, including well-being in older adults [1], depression, anxiety, and sleep quality in adults [2], pain in children and adults [3] and mental and physical health in various populations with different conditions [4]. 

However, few studies have evaluated the potential benefits of laughter therapy as an adjunct to the standard treatment of patients with addictive disorders. Despite Canha’s [5] call for the need to investigate the value of laughter and humour interventions as adjunctive treatments in substance use disorders (SUDs), very little research has been performed in this area to date. To our knowledge, only two studies have explored the effectiveness of laughter therapy in patients with addictive disorders and these were performed in patients with behavioural addictions, not SUDs. These studies conducted in Korea and India among the so-called “smartphone addicts” have shown that laughter therapy is feasible and effective in reducing insomnia [6] and stress [7], and in increasing physical flexibility [6].

The main objective of the present study is to evaluate the benefits of integrative laughter therapy (ILT) on the mental health of patients with addictive disorders. Specifically, we assessed changes in self-esteem, trait anxiety and happiness/satisfaction with life after an ILT intervention in patients treated for addiction at a day hospital.

## 2. Method

### 2.1. Design, Setting and Participants

This was a naturalistic, prospective, longitudinal study carried out within the context of a day hospital (DH). All participants (n = 185) had a current diagnosis of a substance use disorder or gambling disorder and were receiving treatment at the Addictive Behaviours Unit of the Psychiatry Department of the Hospital de la Santa Creu i Sant Pau in Barcelona (Catalonia, northeast Spain). 

### 2.2. Measures

#### 2.2.1. Rosenberg Self-Esteem Scale (RSES) 

The RSES is a 10-item scale designed to assess feelings of self-respect and self-acceptance. The RSES is commonly used to assess overall levels of self-esteem. This scale has shown adequate internal consistency (Cronbach’s alpha coefficient: 0.87) and satisfactory temporal reliability for a two-month interval (Pearson’s correlation coefficient: 0.72) in a Spanish clinical population [8].

#### 2.2.2. The Trait Subscale (STAI-T) of the State-Trait Anxiety Inventory 

The STAI-T is a 20-item instrument designed to measure stable anxiety symptoms. The satisfactory psychometric properties of the STAI-T have been demonstrated in various clinical samples from Spain [9].

#### 2.2.3. Subjective Happiness Scale (SHS) 

The SHS is a 4-item instrument designed to evaluate the overall level of subjective happiness. This scale presents a clear unifactorial structure, adequate internal consistency (Cronbach’s alpha: 0.81), satisfactory temporal reliability for a 6–8-week interval (Pearson correlation coefficient: 0.72) and a satisfactory convergent validity in a wide sample of Spanish adults [10].

#### 2.2.4. Satisfaction with Life Scale (SWLS) 

The SWLS is a short, 5-item instrument widely used to measure the degree of satisfaction with life. A recent study in a representative sample of the Spanish population found that the SWLS presents a unifactorial structure with satisfactory internal consistency (Cronbach’s alpha: 0.88), and the scale was significantly but moderately correlated with the degree of subjective happiness and social support levels [11]. 

#### 2.2.5. Pemberton Happiness Index (PHI) 

The PHI is a comprehensive measure of well-being. The index includes 11 items related to ‘remembered well-being’ (general, hedonic, eudaimonic and social well-being) and 10 items related to ‘experienced well-being’ (positive or negative emotional events that may have occurred the day before). This instrument presents a unifactorial structure, satisfactory internal consistency (Cronbach’s alpha: 0.92) and satisfactory convergent validity in a sample of Spanish adults [12].

### 2.3. Procedure

Patients were recruited through a consecutive, non-probability sampling method at the DH. The initial assessment was performed by the head nurse at the unit. Patients who met the two eligibility criteria (i.e., admission to the DH treatment and age ≥ 18) and accepted to participate in the study were scheduled for the first visit during which all relevant clinical and sociodemographic variables were recorded and the participants completed the self-report measures.

All participants received the standard multicomponent treatment at the DH (treatment as usual; TAU). In addition, they were invited to attend the group ILT sessions (duration: 90 min) offered weekly during the two-month DH treatment regimen (a total of nine ILT sessions). The ILT sessions were held at the facilities of the treatment unit, either the group therapy room or the gymnasium. The workshops were led by a mental health nurse accredited as a laughter therapist.

The nine ILT sessions were designed to ensure that all participants experienced laughter—spontaneously by contagion with other participants or by forcing laughter. All of the sessions had a similar structure, except for differences in the specific theme and objective of the sessions [13,14] (Table 1). Each session had four phases. First, a group activity (e.g., a game) was performed to help the participants adapt to the session and to make them feel comfortable. In these initial group dynamic activities, the aim was not to induce laughter, but rather, through play, to progressively foster participation in the intervention. One concrete example of these games/group dynamic activities is the following: participants were asked to pass an imaginary object to the other participants, but to change the size or temperature of the object using gestures to convey the change in the object. 

The second phase of each session included group dynamic activities targeted at the specific objective for that session (see Table 1). For example, session number 3 included a game in which the participants were given an object and told to look for unusual uses of that object (the more unusual, the better). This game brought on nonsensical situations, thus eliciting laughter from some participants. The third phase was focused on purposefully inducing and exploring laughter through various laughter yoga exercises, such as asking participants to assume one of the usual laughter yoga poses (e.g., laying on the floor, sitting on the floor back to back) and then instructing them to laugh as if the laughter went up from toe to head (like a wave or a current), and finally to laugh loudly when the laughter reached the head. The fourth and final phase at the end of each session involved a brief relaxation exercise after which the group members shared their overall impressions about the session.

Upon completion of the 2-month DH treatment program, the patients were classified according to their attendance at the ILT sessions. Patients who attended ≥80% of the ILT workshops (i.e., 8 or 9 sessions) constituted the experimental group (TAU + ILT group) while those who attended <80% (i.e., 0 to 7 sessions) were considered controls (TAU group). The study was conducted in accordance with the Declaration of Helsinki and its later amendments. The study protocol was approved by the Clinical Research Ethics Committee of the Hospital de la Santa Creu i Sant Pau (approval code: IIBSP-RIS-2018-32). Written informed consent was obtained from all patients included in the study. 

### 2.4. Statistical Analyses

A descriptive analysis of the main sociodemographic, clinical, and treatment-related variables was performed. The results of the mental health scales were also described. Measures of central tendency (mean) and dispersion (standard deviation) were used to describe continuous variables while frequencies and percentages were used for categorical variables. Independent t-tests and chi-square tests were used to check for differences between the groups (TAU vs. TAU + ILT) in baseline characteristics, including sociodemographic and clinical variables (including psychopharmacological treatment) and on the scores obtained on the mental health instruments. Between-group differences in pre-post changes in mental health scale scores were evaluated by repeated measures analysis of the variance (ANOVA) based on a mixed design with a between-subjects factor (“treatment group”) with two levels (TAU and TAU + ILT) and a within-subjects factor (“time factor”) with two levels (i.e., pre- and post-treatment scores). The assumptions about data distribution (i.e., approximate normality of residuals, and homogeneity of variances) were tested and confirmed prior to carrying out the mixed ANOVAs, which were followed by (when appropriate) post hoc Sidak tests to correct for multiple comparisons. All tests of significance were two-tailed, and statistical significance was defined as *p* < 0.05 unless otherwise stated. For mixed ANOVAs, effect sizes were reported as partial η^2^. Small, medium, and large effect sizes correspond to values of partial η^2^ of 0.01, 0.06, and 0.14, respectively [15]. The IBM SPSS Statistics for Windows (Version 24, IBM Corp., Armonk, NY, USA) was used to perform the statistical analyses.

## 3. Results

### 3.1. Sociodemographic and Clinical Characteristics of the Sample

Of the 185 patients enrolled in the study at the start of the DH treatment program, 117 (63.2%) completed the full 2-month program. The remaining 68 patients (36.8%) did not complete the program because of either 1) voluntarily abandonment of the treatment or 2) discharge from the DH prior to completing the treatment (mainly because of relapse or transfer to another treatment centre). Consequently, the final sample consisted of 117 participants (37 women and 80 men). The mean patient age was 47.1 ± 10.2 years (range: 24–70). Most patients (77.4%) were referred to the DH from health care centres located within the city of Barcelona, including 34.8% from our hospital (Hospital de la Santa Creu i Sant Pau). All participants had a confirmed diagnosis of substance use disorder (99.1%) or gambling disorder (0.9%) based on a non-structured clinical interview by the referring psychiatrist (DSM-5 criteria). The distribution of patients according to the main substance or addictive behaviour that prompted the referral was as follows: alcohol (45.3%), cocaine (41.0%), cannabis (5.1%), opioids (3.4%), benzodiazepines (2.6%), amphetamines and derivatives (1.7%), and pathological gambling (0.9%). Of these 117 patients, 99 (84.6%) attended ≥80% of the ILT sessions (8.8 ± 0.4 sessions, range: 8-9) while 18 (15.4%) attended <80% of the sessions (1.4 ± 1.5, range: 0–5).

At baseline, there were no significant between-group differences (Table 2) in sociodemographic or clinical variables, except for sex (males accounted for a larger proportion of the TAU group).

As Table 3 shows, there were no statistically significant differences between the groups on any of the mental health scales administered pre-treatment.

### 3.2. Changes in Self-Esteem from Baseline to Treatment Finalization by Group

The results of the repeated measures ANOVA on RSES scores (Table 4) revealed the following significant effects: Time effect [F(1, 115) = 8.656; *p* = 0.004; partial η^2^ = 0.070], and group-by-time interaction [F(1, 115) = 6.744; *p* = 0.011; partial η^2^ = 0.055]. By contrast, the group effect was not significant [F(1, 115) *=* 0.344; *p* = 0.559; partial η^2^ = 0.003]. 

These results show significant between-group differences in the pre-post temporal change in RSES scores. Although the mean RSES score increased in both groups, the increase was significantly greater in the combined treatment group (TAU + ILT) compared to the TAU group.

### 3.3. Changes in Trait Anxiety from Baseline to Treatment Finalization by Group

The results of the repeated measures ANOVA presented in Table 5 show that the time effect [F(1, 115) = 6.011; *p =* 0.016; partial η^2^ = 0.050] and the group-by-time interaction [F(1, 115) = 4.930; *p* = 0.028; partial η^2^ = 0.041] were both statistically significant for the STAI-T scores of the State-Trait Anxiety Questionnaire. However, the group effect was not significant: [F(1, 115) = 3.564; *p* = 0.062; partial η^2^ = 0.030].

These results show significant between-group differences in the temporal changes of the STAI-T subscale scores. Although the scores on this subscale decreased in both groups over time, this decrease was significantly greater in the TAU + ILT group. 

### 3.4. Changes in Happiness from Baseline to Treatment Finalization by Group

The results of the repeated measures ANOVA on the SHS scores (Table 6) revealed the following significant effects: time effect [F(1, 115) = 10.747; *p* = 0.001; partial η^2^ = 0.085], and group-by-time interaction [F(1, 115) = 4.662; *p* = 0.033; partial η^2^ = 0.033]. By contrast, the group effect was not significant [F(1, 115) = 1.469; *p* = 0.228; partial η^2^ = 0.013].

These results show significant between-group differences in the pre-post temporal change in SHS scores. Although the scores on this scale increased in both groups after completion of treatment, the increase was significantly more marked in the TAU+ILT group.

Similar results were obtained (Table 6) for SWLS scores, with both time [F(1,115) = 5.523; p = 0.020; partial η^2^ = 0.046] and group-by-time interaction [F(1, 115) = 9.626; *p* = 0.002; partial η^2^ = 0.077] effects reaching statistical significance; however, the group effect was not significant [F(1, 115) = 2.487; *p* = 0.118; partial η^2^ = 0.021]. These results demonstrate significant between-group differences in the pre-post changes in SWLS scores. In the TAU group, there was a slight decrease in SWLS scores; by contrast, the TAU+ILT showed a marked increase in SWLS scores. 

Regarding PHI scores (Table 6), the repeated measures ANOVA revealed a statistically significant time effect [F(1, 115) = 10.013; *p* = 0.019; partial η^2^ = 0.047], group-by-time interaction [F(1,115) = 13.944; *p* = 0.006; partial η^2^ = 0.065], and group effect [F(1, 115) = 5.000; *p* = 0.027; partial η^2^ = 0.042]. This last effect was due to a large difference between treatment groups (favouring the TAU + ILT group) in post-treatment values (mean difference between groups = 1.556, Sidak-adjusted *p*-value = 0.0004). These results demonstrate significant between-group differences in the temporal changes in PHI scores. There was a small decrease in the PHI score in the TAU group over time, but a moderate increase in the TAU + ILT group. 

### 3.5. Sensitivity Analysis

A sensitivity analysis revealed that creating two equally sized groups (the TAU + ILT group was reduced to 18 participants by randomly selecting 18 of the 99 patients) did not substantively change the results (data not shown); the same statistically significant group-by-time interaction effects were obtained, except for SHS, which did not reach the threshold for statistical significance.

## 4. Discussion

To our knowledge, this is the first study to evaluate the effects of an integrative laughter therapy module on levels of self-esteem, anxiety, and happiness in patients with addictive disorders treated at a day hospital. The findings of the present study are, on the whole, positive and promising. Our results demonstrate the viability of incorporating an ILT workshop or module into the therapeutic arsenal of the standard multicomponent program used to treat individuals with addictions in the context of a DH. These findings show that participation in an ILT module enhances the effectiveness of the standard DH treatment in terms of several non-consumption outcomes, as evidenced by the significant association between ILT participation and greater improvement (versus DH alone) in positive and negative mental health symptoms. Effect sizes for self-esteem, anxiety and happiness were mostly in the low-to-medium range, as determined by the benchmarks suggested by Cohen [15].

In terms of self-esteem, our results are consistent with those reported by Rudnick et al. [16], who evaluated a humour-related intervention (stand-up comedy training facilitated by a professional comedian) in patients with mental disorders. That study found a significant positive impact on self-esteem after the intervention. 

Although we observed a statistically significant decrease in mean anxiety scores in both groups, the improvement was more marked in the TAU + ILT group. These results are consistent with several studies that have found a decrease in anxiety levels after humour-based interventions. Ghodsbin and colleagues [17] evaluated a group of older people, finding a larger decrease in anxiety and insomnia levels in participants who completed the laughter therapy program. Yu and Kim [18] assessed stress response and pain levels in military personnel who received laughter therapy for back pain on three consecutive days, finding that post-intervention anxiety and depression levels were significantly lower in the experimental group versus controls. Finally, Hatzipapas and collaborators [19] showed that laughter therapy was effective in reducing anxiety levels in community care workers who worked with HIV-affected families. 

In the present study, patients in the TAU + ILT group achieved a greater increase in life satisfaction scores, a finding that contrasts with the aforementioned study by Rudnick and colleagues [16], who found no statistically significant differences between groups in terms of recent satisfaction with life (assessed quantitatively using the Satisfaction with Recent Life Scale). Nevertheless, the qualitative results of that study suggested a post-intervention increase in patient-reported life satisfaction. 

Our results also coincide with those reported by Tse et al. [20] who evaluated a group of older adults with chronic pain who received a humour therapy intervention. The authors found significant improvements in satisfaction with life (assessed by means of the Life Satisfaction Index-A scale) in the experimental group. In that same study, the experimental group also achieved a significantly greater improvement in levels of perceived happiness (measured with the SHS) compared to the controls. These results are consistent with our findings, in which SHS scores increased significantly more in the TAU + ILT group.

### 4.1. Study Limitations

The present study has several limitations, including those inherent to any study using a quasi-experimental, pre-post design without a fully comparable control group in the context of routine clinical practice in a single centre. An important design-related limitation is the non-random allocation of participants to the two treatment conditions; as a result, we cannot be certain that the differences observed were solely attributable to the addition of ILT intervention. An additional limitation of the study is the lack of balance between the number of participants in each group. Nevertheless, a sensitivity analysis (using equally sized samples) revealed no substantial changes in the direction or significance of most of the results obtained. Another limitation is that the study’s results—obtained in the context of routine clinical practice in a day hospital—cannot be generalized to patients with addictive disorders treated in other types of centres (outpatient or inpatient), which may administer more or less intense therapeutic approaches. Our selection of this study design and treatment setting was to find equilibrium between ethical and practical considerations (which often makes it difficult or even impossible to randomly assign patients to different treatment conditions in the real world of clinical setting) and the feasibility of the study itself. In any case, despite the non-random treatment allocation, the two groups (TAU and TAU + ILT) come from the same patient population given the lack of statistically significant differences in sociodemographic variables (except sex) between the groups. Moreover, the effect size (φ = 0.188) of this single difference did not reach the recommended minimum value to be considered a ‘practically’ significant effect for social science data [21]. Finally, this study of the effectiveness of the ILT workshop included only patients who completed the DH treatment program and all mental health scales administered pre- and post-intervention, thus excluding patients who did not complete all the pre- and post-treatment evaluations (in the latter case, mainly because of not having completed the treatment in the day hospital regime). This type of analysis (i.e., complete cases analysis), although still prevalent in clinical trials published in prestigious medical journals [22,23], can have non-negligible repercussions, including loss of statistical power (because of the reduction in sample size) as well as the potential introduction of unacceptable biases. For this reason, these findings should be interpreted cautiously. In any case, complete cases analysis is an appropriate analytical approach in exploratory studies such as the present study or in the initial phases of the evaluation of the effectiveness of an intervention [24]. 

### 4.2. Future Implications

Despite the aforementioned study limitations, mainly attributable to the quasi-experimental design, the findings of the present study suggest that a humour-based therapeutic strategy may improve the mental well-being of patients with addiction problems. Therefore, the incorporation of an ILT module to treatment-as-usual in a day hospital regimen for addictive behaviours could significantly increase the effectiveness of standard care, improving the recovery of patients with addiction problems. 

Further research is warranted using randomized clinical trials in the process of introducing ILT workshops as an adjunct to the standard treatment of patients with addictive disorders.

## 5. Conclusions

The current study shows that the addition of an integrative laughter therapy module to the standard treatment regimen in a day hospital for addictive disorders may lead to a greater improvement in measures of self-esteem, anxiety and happiness compared to treatment as usual.

## Figures and Tables

**Table 1:** Titles and objectives of the integrative laughter workshop sessions.

Session Number	Session Title	Main Objective
1	Everybody as a system	Group cohesion: competition and collaboration
2	I am and I can also be	Self-esteem, self-concept, and motivation
3	They are looking at me	Creativity with humour and resilience
4	Internal barriers	Relaxing and unblocking
5	I care for myself and you	Confidence and communication
6	Living	Senses and attention
7	Two more minutes	Realistic positivism
8	Exciting music	Managing emotions
9	My rosebush	Emotional intelligence

**Table 2:** Sociodemographic and clinical characteristics.

		TAU Group (*n* = 18)		TAU + ILT Group (*n* = 99)		Test	*p*
		n	%	n	%		
*Sex*						χ^2^(1) = 4.140	0.042
Female		2	11.1	35	35.4		
Male		16	88.9	64	64.6		
*Age* (mean ± SD)		49.4 ± 11.1		46.6 ± 10.0		t(115)= 1.073	0.286
*Main substance*						χ^2^(6) = 7.271	0.296
Alcohol		9	50.0	44	44.4		
Cocaine		5	27.8	42	43.4		
Cannabis		2	11.1	4	4.0		
Opioids		2	11.1	2	2.0		
Benzodiazepines		0	0	3	3.0		
Amphetamines		0	0	2	2.0		
Gambling		0	0	1	1.0		
*HIV*						χ^2^(1) = 0.995	0.319
No		16	88.9	94	94.9		
Yes		2	11.1	5	5.1		
*HCV*						χ^2^(1) = 0.665	0.415
No		15	83.3	89	89.9		
Yes		3	16.7	10	10.1		
*Dual pathology*						χ^2^(1) = 0.056	0.813
No		10	55.6	52	52.5		
Yes		8	44.4	47	47.5		
*Treatment with disulfiram*						χ^2^(1) = 0.322	0.571
No		8	44.4	37	37.4		
Yes		10	55.6	62	62.6		
*Treatment with methadone*						χ^2^(1) = 0.610	0.435
No		16	88.9	93	93.9		
Yes		2	11.1	6	6.1		
*Treatment with neuroleptics*						χ^2^(1) = 0.002	0.965
No		13	72.2	71	71.7		
Yes		5	27.8	28	28.3		
*Antidepressive treatment*						χ^2^(1) = 0.823	0.364
No		11	61.1	49	49.5		
Yes		7	38.9	50	50.5		
*Treatment with mood stabilizers*						χ^2^(1) = 0.104	0.747
No		16	94.1	90	91.8		
Yes		1	5.9	8	8.2		

TAU: treatment as usual; ILT: integrative laughter therapy; SD: standard deviation; HIV: human immunodeficiency virus; HCV: hepatitis C virus.

**Table 3:** Mean scores on the mental health scales administered prior to treatment initiation.

	TAU Group (*n* = 18)	TAU + ILT Group (*n* = 99)	Test	*p*
*Self-esteem*				
RSES	28.61 ± 6.42	27.67 ± 5.69	t(115) = 0.635	0.527
*Anxiety*				
STAI-T	7.50 ± 1.86	7.19 ± 1.87	t(115) = 0.643	0.521
*Happiness*				
SHS	3.83 ± 1.06	3.86 ± 1.21	t(115) = −0.083	0.934
SWLS	17.73 ± 6.42	17.68 ± 6.42	t(115) = 0.033	0.974
PHI	5.36 ± 1.77	5.56 ± 1.93	t(115) = −0.411	0.682

Data expressed as means ± SD. TAU: treatment as usual; ILT: integrative laughter therapy; RSES: Rosenberg Self-Esteem Scale; STAI-T: Trait Subscale of the State-Trait Anxiety Inventory; SHS: Subjective Happiness Scale; SWLS: Satisfaction with Life Scale; PHI: Pemberton Happiness Index.

**Table 4:** Scores on the Rosenberg Self-esteem Scale (RSES) at the start and completion of the day hospital treatment by group.

	TAU Group (*n* = 18)		TAU + ILT Group (*n* = 99)		Time Effect	Group-by-Time Effect	Group Effect
	Start	End	Start	End			
*Self-esteem*							
RSES	28.61 ± 6.42	28.83 ± 4.77	27.67 ± 5.69	31.23 ± 5.13	*p* = 0.004	*p* = 0.011	*p* = 0.559

Data expressed as means ± SD. TAU: treatment as usual; ILT: integrative laughter therapy.

**Table 5:** Scores on the Trait-Anxiety Subscale (STAI-T) at the start and completion of the day hospital treatment by group.

	TAU Group (*n* = 18)		TAU Group + ILT (*n* = 99)		Time Effect	Group-by-Time Effect	Group Effect
	Start	End	Start	End			
*Anxiety*							
STAI-T	7.50 ± 1.86	7.44 ± 1.58	7.19 ± 1.87	6.07 ± 2.15	*p* = 0.016	*p* = 0.028	*p* = 0.062

Data expressed as means ± SD. TAU: treatment as usual; ILT: integrative laughter therapy.

**Table 6:** Scores on three measures of happiness at the start and completion of the day hospital treatment by group.

	TAU Group (*n* = 18)		TAU + ILT Group (*n* = 99)		Time Effect	Group-by-Time Effect	Group Effect
	Start	End	Start	End			
*Happiness scales*							
SHS	3.83 ± 1.06	3.99 ± 0.80	3.86 ± 1.21	4.60 ± 1.17	*p* = 0.001	*p* = 0.033	*p* = 0.228
SWLS	17.73 ± 6.42	17.19 ± 4.43	17.68 ± 6.42	21.62 ± 5.99	*p* = 0.020	*p* = 0.002	*p* = 0.180
PHI	5.36 ± 1.77	5.26 ± 1.78	5.56 ± 1.93	6.81 ± 1.66	*p* = 0.019	*p* = 0.006	*p* = 0.027

Data expressed as means ± SD. TAU: treatment as usual; ILT: integrative laughter therapy; SHS: Subjective Happiness Scale. SWLS: Satisfaction with Life Scale. PHI: Pemberton Happiness Index.
